# Galaxy ASIST: A web-based platform for mapping and assessment of global standards of antimicrobial susceptibility: A case study in *Acinetobacter baumannii* genomes

**DOI:** 10.3389/fmicb.2022.1041847

**Published:** 2023-01-27

**Authors:** Tina Sharma, Rakesh Kumar, Jasmeer Singh Kalra, Shreya Singh, Gurpreet Singh Bhalla, Anshu Bhardwaj

**Affiliations:** ^1^Bioinformatics Centre, CSIR-Institute of Microbial Technology, Chandigarh, India; ^2^Academy of Scientific and Innovative Research (AcSIR), Ghaziabad, India; ^3^Department of Biotechnology, Delhi Technological University, Delhi, India; ^4^CSIR-Institute of Microbial Technology, Chandigarh, India

**Keywords:** Galaxy, EUCAST/CLSI guidelines, AMR, *Acinetobacter baumannii*, antimicrobial susceptibility, whole genome sequencing, Minimum inhibitory concentration

## Abstract

**Introduction:**

Antimicrobial susceptibility testing (AST) is used to determine the susceptibility of an organism to antibiotics. The determination of susceptibility is based on MIC breakpoints and is provided by EUCAST and CLSI. Likewise, phenotypic classification criteria developed by CDC/ECDC are used for the classification of pathogens into susceptible, multidrug-resistant, extremely drug-resistant, or totally drug-resistant categories. Whole-genome sequencing (WGS)-based diagnosis is now supplementing existing gold-standard microbiology methods for rapid and more precise AST, and therefore, EUCAST recommended quality criteria to assess whole-genome sequence for reporting the same. In this study, these three global standards, MIC breakpoints, phenotypic classification, and genome quality, are applied to the largest publicly available data for Acinetobacter baumannii (AB), the most critical priority pathogen identified by WHO.

**Materials and Methods:**

The drug sensitivity profile and genomes for isolates of AB were obtained from PATRIC and evaluated with respect to AST standards (CLSI and EUCAST). Whole genome quality assessment and antimicrobial resistance mapping is performed with QUAST and ABRicate, respectively. Four in-house methods are developed for mapping standards and are integrated into a Galaxy workflow based system, Galaxy-ASIST. Analysis of the extent of agreement between CLSI 2022 and EUCAST 2022 for antibiotics was carried out using Cohen’s kappa statistics.

**Results and Discussion:**

An automated pipeline, Galaxy-ASIST, is designed and developed for the characterization of clinical isolates based on these standards. Evaluation of over 6,500 AB strains using Galaxy-ASIST indicated that only 10% of the publicly available datasets have metadata to implement these standards. Furthermore, given that CLSI and EUCAST have different MIC breakpoints, discrepancies are observed in the classification of resistant and susceptible isolates following these standards. It is, therefore, imperative that platforms are developed that allow the evaluation of ever increasing phenotypic and genome sequence datasets for AST. Galaxy-ASIST offers a centralized repository and a structured metadata architecture to provide a single globally acceptable framework for AST profiling of clinical isolates based on global standards. The platform also offers subsequent fine mapping of antimicrobial-resistant determinants. Galaxy-ASIST is freely available at https://ab-openlab.csir.res.in/asist.

## Introduction

Antimicrobial resistance (AMR) is a major public health concern (Price, 2016). Toward this, rapid molecular diagnostic tests (RDTs) are being developed not only to detect bacteria in clinical samples but also to determine antibiotic susceptibility with rapid turnaround time (TAT) (Glover et al., 2018). Antimicrobial susceptibility testing (AST) is performed to classify a strain as resistant or susceptible to antibiotics Bayot and Bragg (2022). The AST is based on MIC breakpoints provided by the Clinical Laboratory Standards Institute (CLSI) or European Committee on Antimicrobial Susceptibility Testing (EUCAST) (Jenkins and Jerris, 2011), and automated platforms are available for the same. However, the methods for detection of resistance are not yet automated. Similarly, a global criterion to classify strains as multiple or extremely or totally drug-resistant is established by the European Centre for Disease Prevention and Control (ECDC) and Centre for Disease Control and Prevention (CDC). These laboratory procedures require culturing of bacteria and can take up to 48 h or longer to obtain results (Pereira and Goldani, 2019). Whole-genome sequencing (WGS)-based diagnosis can supplement these existing gold-standard microbiology methods not only for rapid TAT but also to discover new resistance markers to aid in the selection of appropriate treatment regimen (Pereira and Goldani, 2019). Given the decreasing costs and high resolution provided by WGS (Castillo-Ramírez and Graña-Miraglia, 2019), a large number of pathogenic organisms are sequenced to improve the quality of molecular diagnostics, therapeutics, and surveillance (Köser et al., 2014). In recent years, there has been an unprecedented increase in high-throughput DNA sequencing of genomes and metagenomes toward bacterial typing, molecular epidemiology investigations, and in-depth pathogenic studies (Kwong et al., 2015). In addition to the identification of new markers to detect microbial infections, WGS can provide insight into the role of genomic variation in strain evolution and its impact on transmission in real time (Neerman et al., 2019).

To standardize the use of genomic-based AST, EUCAST has recommended standards for genome quality for reporting antibiotic susceptibility. Standardization and digitization of microbiological and molecular data and analysis reports are critical for a modern microbiology laboratory to make optimal use of technology advancements for patient-centered care (Egli et al., 2020). It is imperative that standard vocabulary and metadata are defined and used for ease of access, analysis, and utilization of these data for quick and actionable observations in healthcare settings. The challenge is that more and more research laboratories and clinics are generating a vast amount of genome sequence datasets from clinical, environmental, and animal studies and gaps in reporting standards are leading to poor convergence in result outcomes. As mentioned earlier, different stages of strain characterization are based on different global standards, and therefore, in this study, we focused on the convergence of standards for MIC breakpoints by EUCAST and CLSI, phenotypic classification criteria by CDC and ECDC, and standards for assessing the quality of whole-genome sequence for reporting antimicrobial susceptibility by EUCAST for *Acinetobacter baumannii* (AB), as it is known to cause a plethora of diseases, especially in the hospital setting. Moreover, it can survive in the environment and is resistant to multiple classes of antimicrobial agents and labeled as the most critical priority pathogen by the WHO (WHO, 2017). Recent reports indicate that the rate of carbapenem-resistant *Acinetobacter* cases has increased by 35% from 2019 to 2020 (CDC, 2022). The results from Antimicrobial Testing Leadership and Surveillance (ATLAS) program, 2012-2019 showed significant differences in the prevalence of carbapenem resistant AB among countries in the Asia-Pacific region (Lee et al., 2022). Therefore, in this study, Galaxy-ASIST is designed to integrate global standards in an automated pipeline and is used to assess the publicly available datasets for AB.

The Galaxy-ASIST platform is designed to reduce the complexity of data analysis and offers a user-friendly interface for mapping global standards for reporting antimicrobial susceptibility profiles. In addition, it also offers utilities for integrating data for antimicrobial resistance or virulence genes for the strains evaluated using the platform. A comparison of strains classified as resistant or susceptible with CLSI and EUCAST standards is also performed to report the observations from publicly available resources on AB. Galaxy-ASIST is developed as a Galaxy workflow system, as it offers immense scalability and modularity in terms of creating custom workflows. In addition to performing mapping with the three global standards, the users have access to the wide array of analysis offered as part of the Galaxy workflow system. Despite the fact that Galaxy-ASIST is tested on AB as of now, it can be easily extended to include any pathogen class.

## Methods

### Standard definitions

**Figure 1:**
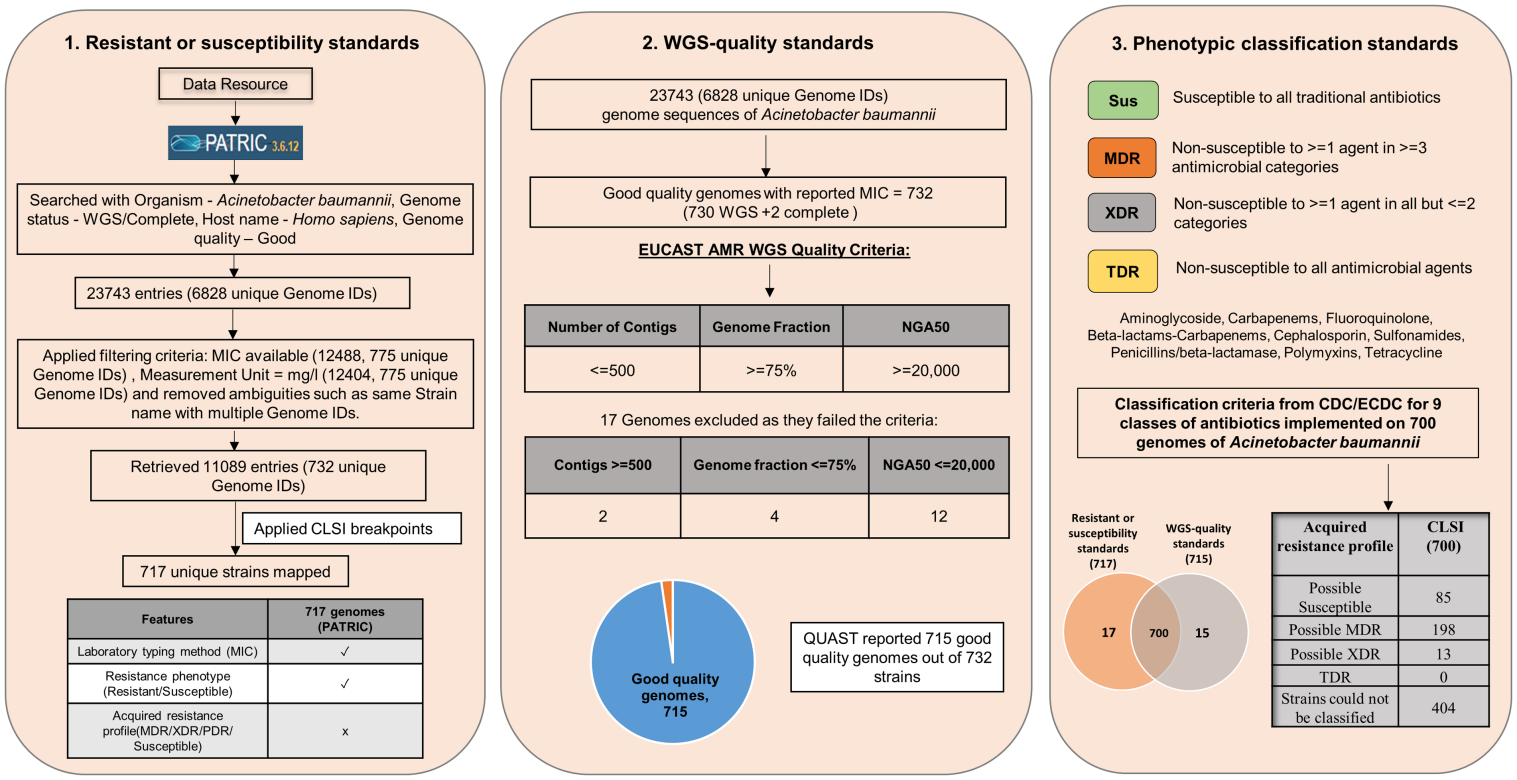
Schematic workflow of implementing global standards to WGS-inferred AST (resistant or susceptibility standards, WGS-quality standards, and Phenotypic classification standards).

This study is focused on the convergence of standards for MIC breakpoints, standards for assessing the quality of whole-genome sequence for reporting antimicrobial susceptibility, and phenotypic classification criteria with *Acinetobacter baumannii* (AB), as the case study (for standards refer to Supplementary File S1). The schematic workflow of implementing global standards to WGS-inferred AST is given in Figure 1.

### Data mining for drug sensitivity profile of clinical isolates

**Figure 2:**
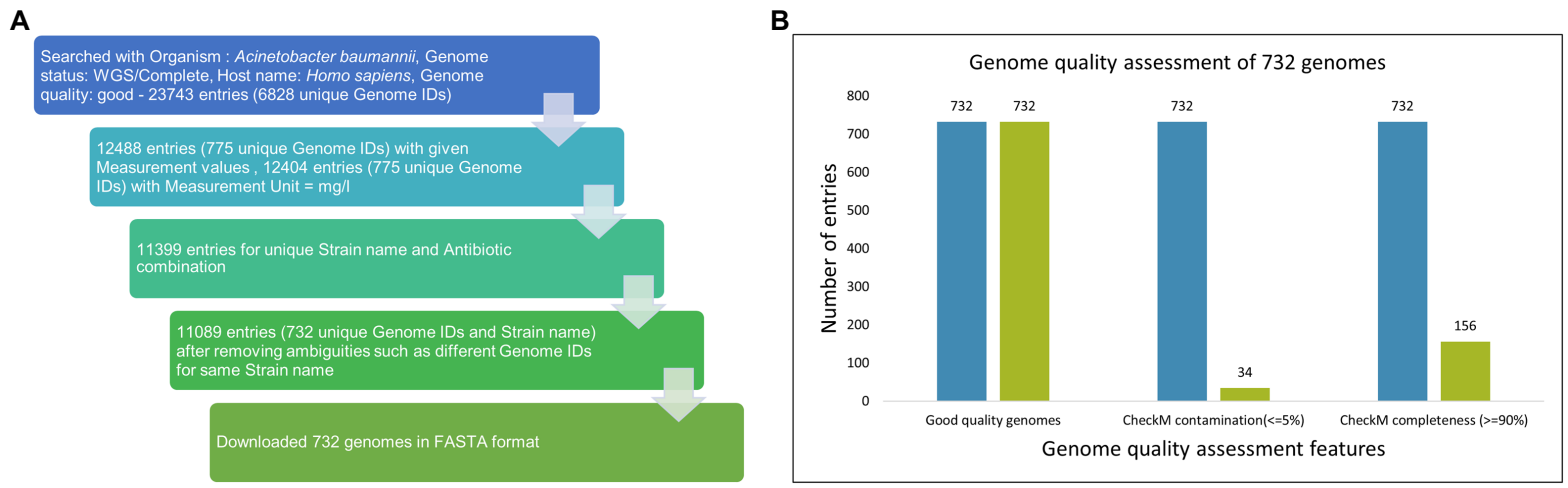
**(A)** Data mining from PATRIC. The drug sensitivity profile for isolates of AB (till 1 July 2022) were obtained from PATRIC (14) using keyword search with “*Acinetobacter baumannii*” and resulted in 23,743 entries. Several filters, namely Host: *Homo sapiens;* Genome quality: Good; Genome status: WGS/Complete, were applied on this dataset, and incomplete entries with no MIC values and duplicate entries were removed. **(B)** Among 732 good quality genomes (as defined by PATRIC), CheckM contamination (≤5%) is reported for 34 and CheckM completeness (>=90%) is reported for 156 genomes.

The drug sensitivity profile for isolates of AB (till 1 July 2022) was obtained from PATRIC (Davis et al., 2020) using a keyword search with “*Acinetobacter baumannii*” which resulted in 23,743 entries. Several filters, namely Host: *Homo sapiens;* Genome quality: Good; Genome status: WGS/Complete, were applied to this dataset, and incomplete entries with no MIC values and duplicate entries were removed (Figure 2A). The data are provided in Supplementary File S2.

### Strain selection based on international standards

The isolates (with MIC) obtained from PATRIC are classified into susceptible and resistant based on breakpoints from CLSI and EUCAST for antimicrobial susceptibility testing (Supplementary File S4; Magiorakos et al., 2012). The results from this classification are then used for further classification by ECDC and CDC for strain classification based on nine antimicrobial categories with 22 antibiotics (Supplementary File S1). Two *in-house* programs are developed based on these classification systems and applied to clinical isolates of AB for classification into susceptible and resistant isolates and subsequently for phenotypic classification into susceptible, MDR, XDR, and TDR. Genome sequences of isolates classified by CLSI as sensitive or resistant are subjected to quality metrics as defined by EUCAST. For the same, QUAST 5.2.0 is used with the reference genome of AB, that is, ATCC 17978. The statistics obtained from QUAST for EUCAST standard mapping used in this study are the total number of contigs (<=500), genome fraction (>=75%), and NGA50 (>=20,000). QUAST can take fasta files as input (both whole genomes and contigs) and gives genome quality metrics as output. Analysis of the extent of agreement between CLSI 2022 and EUCAST 2022 for antibiotics was carried out using Cohen’s kappa statistics (Viera and Garrett, 2005).

### Integration of tools in Galaxy-ASIST

Four in-house methods are developed and integrated into Galaxy-ASIST to support standards mapping and integration of output from these standards mapping programs as a single consolidated output: ASIST_CLSI_Breakpoint, ASIST_Phenotypic_Classification, ASIST_Automate, and ASIST_Integrator. In addition, QUAST and ABRicate are also available on the Galaxy server. (Gurevich et al., 2013; Seemann, 2021; Galaxy Community, 2022).

The ASIST_CLSI_Breakpoint tool categorizes isolates into resistance and susceptible categories based on the CLSI:2022 breakpoints (nine classes with 25 antibiotics; CLSI, 2022). This program takes input as a csv file with column headings–Strain name, Antibiotics, MIC, and Unit. The CLSI:2022 breakpoint file is already embedded in the system. The platform also provides an option for user defined MIC breakpoints as the breakpoints evolve over time. The output of this program annotates the strains into resistant, susceptible, or intermediate depending on their MIC comparison with CLSI MIC breakpoints. In cases where the strain could not be classified based on MIC values, the program indicates the same as “strain could not be classified” and “NA” when MIC (for any given antibiotic) is not available for that particular strain.

The ASIST_Phenotypic_Classification tool categorizes the strains as “Possible susceptible,” “Possible multidrug resistant,” “Extremely drug resistant,” “Totally drug resistant,” and “Strain could not be classified” [in the following format: (“Number of antibiotics categories count as Intermediate” | “Number of antibiotics categories count as NA”)]. The ASIST_Phenotypic_Classification tool takes the output of the ASIST_CLSI_Breakpoint tool in a csv format and classifies the strains according to the CDC/ECDC standards.

To provide a user-friendly interface, ASIST_Automate is developed as an in-house pipeline for Galaxy-ASIST. ASIST_Automate can take three input files that are required by ASIST_CLSI_Breakpoint, ASIST_Phenotypic_Classification, and QUAST and generate an integrated output from all these programs.

To provide information on antimicrobial-resistant genes in strains that qualify all the standards, the output of ABRicate can also be integrated with consolidated output from QUAST, ASIST_CLSI_Breakpoint, ASIST_Phenotypic_Classification, and ABRicate in Galaxy-ASIST. This can be done using the ASIST_Integrator module. The current modules available in Galaxy-ASIST are listed in Table 1.

**Table 1:** Galaxy-ASIST toolkit.

**S. no**	**Tool name**	**Description**
1.	ASIST_CLSI_Breakpoint	CLSI MIC breakpoint profile
2.	ASIST_Phenotypic_Classification	Phenotypic classification
3.	ASIST_Automate	Generates an integrated output of all the above programs based on three input files
4.	ASIST_Integrator	Generates a consolidated result of all the programs based on their output files
5.	QUAST	WGS quality assessment
6.	ABRicate	Mapping antimicrobial resistance genes

### Workflows

The advantage of using Galaxy for standards mapping is the scalability of the system that allows users to generate custom workflows depending on their needs. To provide a glimpse of what kind of workflows may be generated, a sample workflow is provided in Galaxy-ASIST. In this workflow, the user can provide two inputs–csv file with MIC values (column names: Strain name, Antibiotics, MIC, and Unit) and the corresponding genome sequence of the strains as fasta file(s). The workflow performs breakpoint mapping and phenotypic classification on the csv file including standard mapping by QUAST. The fasta files are also used for AMR determinant mapping by ABRicate. The workflow integrates outputs from all these programs and provides the same as a consolidated output file.

## Results

Publicly available MIC and associated genome sequence data for *Acinetobacter baumannii* obtained from PATRIC were filtered on several parameters (see Methods). Based on the filtering criteria applied, drug sensitivity profiles and genome sequences of 732 unique isolates were obtained against 44 antibiotics with five in combination (available with MIC in mg/L). These data are used for subsequent mapping to three global standards and for the determination of antimicrobial-resistant determinants.

### Mapping to MIC breakpoint standards

**Figure 3:**
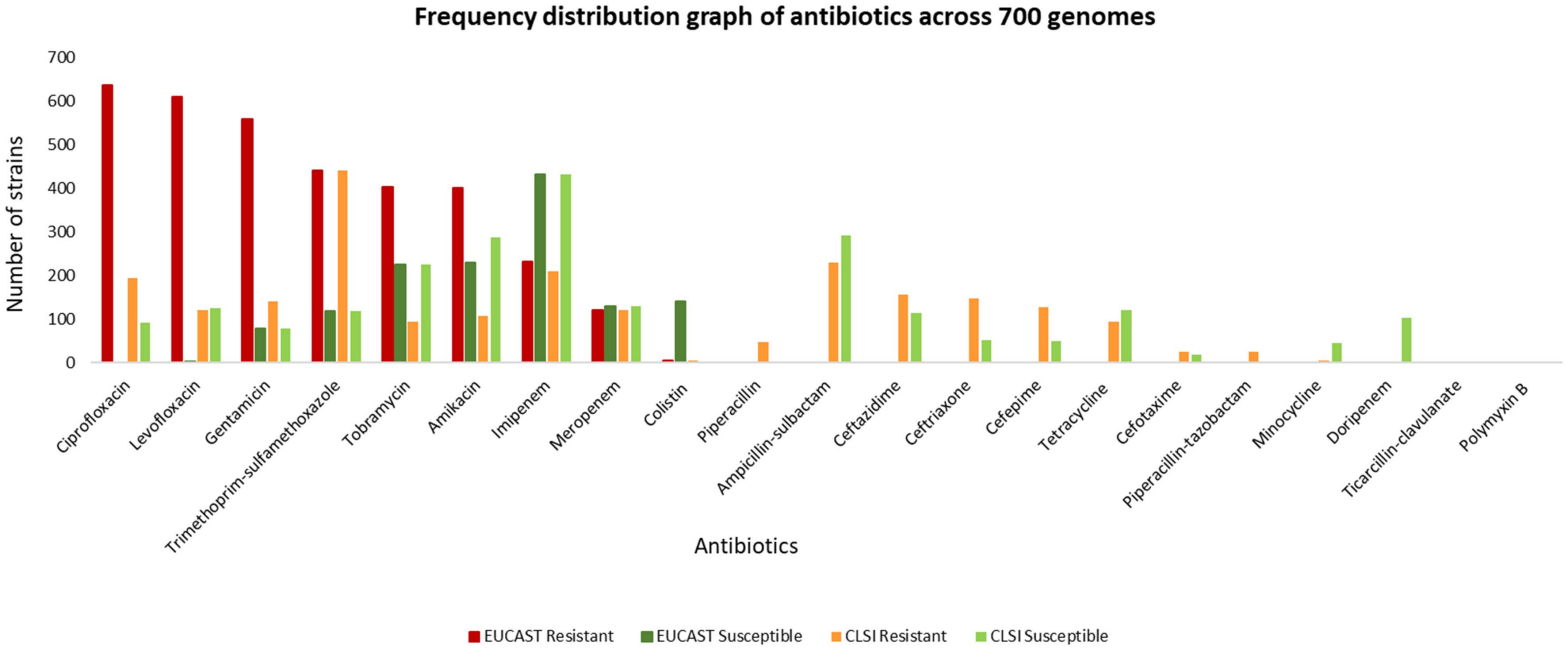
Frequency distribution graph of antibiotics across 700 genomes.

The MIC of 732 unique isolates was compared with CLSI breakpoints for the classification of isolates as susceptible and resistant. Based on this comparison, the ASIST_CLSI_Breakpoint program reports classification of 717 clinical isolates (as 15 entries were of tigecycline for which breakpoints are not provided in the CLSI breakpoints). The MICs for 732 isolates were also compared with EUCAST (EUCAST: 2022) breakpoints (EUCAST, 2022). It was observed that over 400 clinical isolates are resistant to ciprofloxacin, levofloxacin, gentamicin and trimethoprim-sulfamethoxazole (Figure 3, data provided in Supplementary File S2).

**Figure 4:**
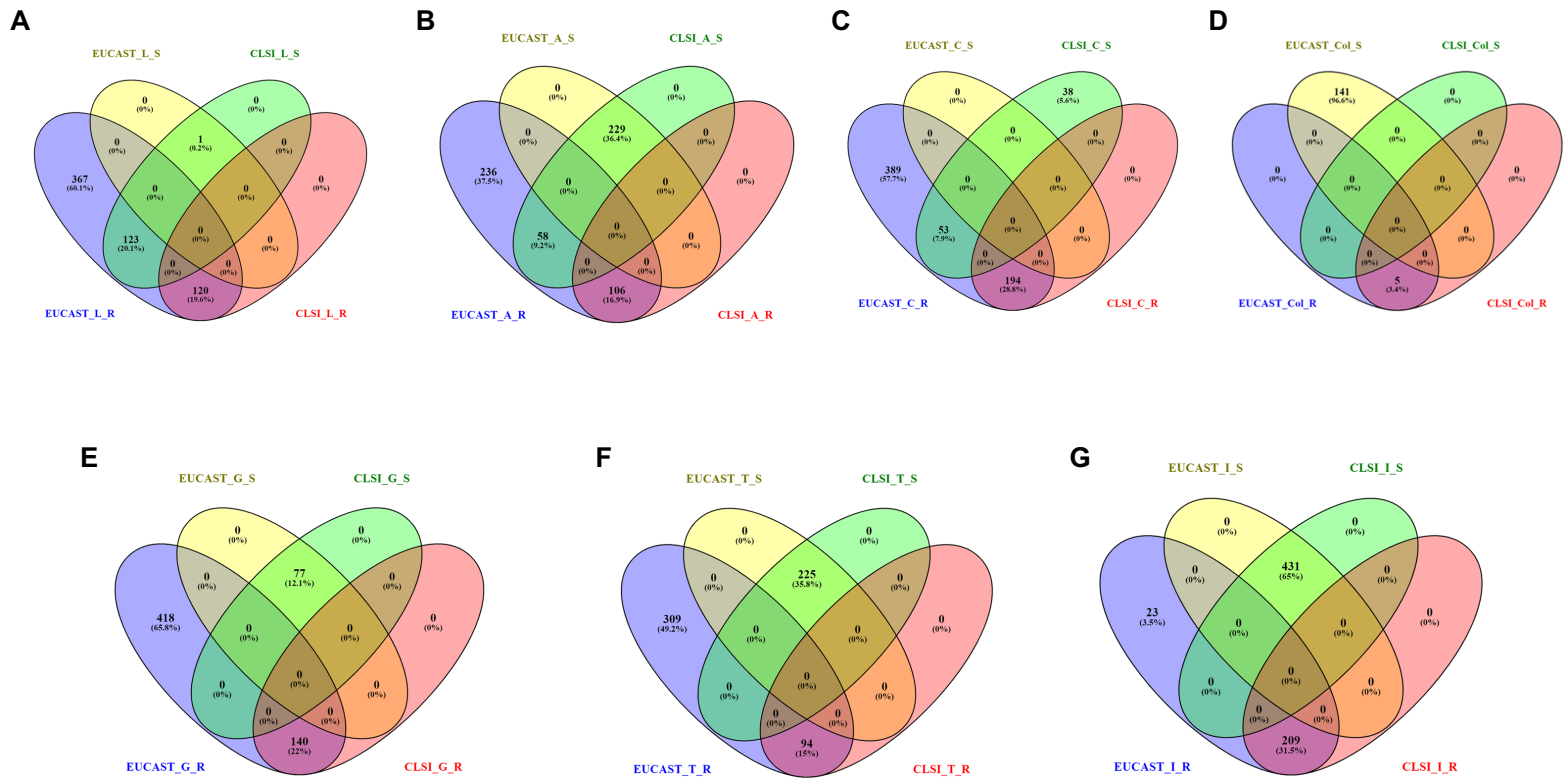
Resistant (R) and susceptible (S) classification by CLSI/EUCAST for the following antibiotics: **(A)** levofloxacin, **(B)** amikacin, **(C)** ciprofloxacin, **(D)** colistin, **(E)** gentamicin, **(F)** tobramycin, and **(G)** imipenem.

On comparing the classification for antibiotics (present in the CLSI breakpoint standard document) performed by CLSI and EUCAST, it is observed that there are significant differences in the interpretation criterion of EUCAST and CLSI. For example, in the case of levofloxacin, 123 isolates are categorized as susceptible by CLSI but resistant by EUCAST, whereas 367 isolates are classified only by EUCAST. Similarly for amikacin, 58 isolates are classified as susceptible and resistant, respectively, by CLSI and EUCAST, and 236 isolates are classified only by EUCAST as resistant. In the case of ciprofloxacin, 53 isolates are mapped as susceptible by CLSI but resistant by EUCAST, and a total of 389 isolates are classified only by EUCAST. In this case, 38 isolates were classified only by CLSI. For colistin, 141 strains are defined as susceptible by EUCAST but remain unclassified by CLSI. In some cases such as gentamicin (418), tobramycin (309), and imipenem (23), there is no classification by CLSI but is categorized as resistant by EUCAST (Figure 4, Supplementary File S2).

Analysis of the extent of agreement between CLSI 2022 and EUCAST 2022 for levofloxacin, amikacin, and ciprofloxacin was carried out using Cohen’s kappa statistics. In the case of levofloxacin, there was slight agreement (*κ* = 0.015) between the AST using CLSI and EUCAST. In the case of ciprofloxacin, there was poor agreement (*κ* = 0) while substantial agreement (*κ* = 0.68) is observed for amikacin.

### Genome quality metrics and standard mapping

**Figure 5:**
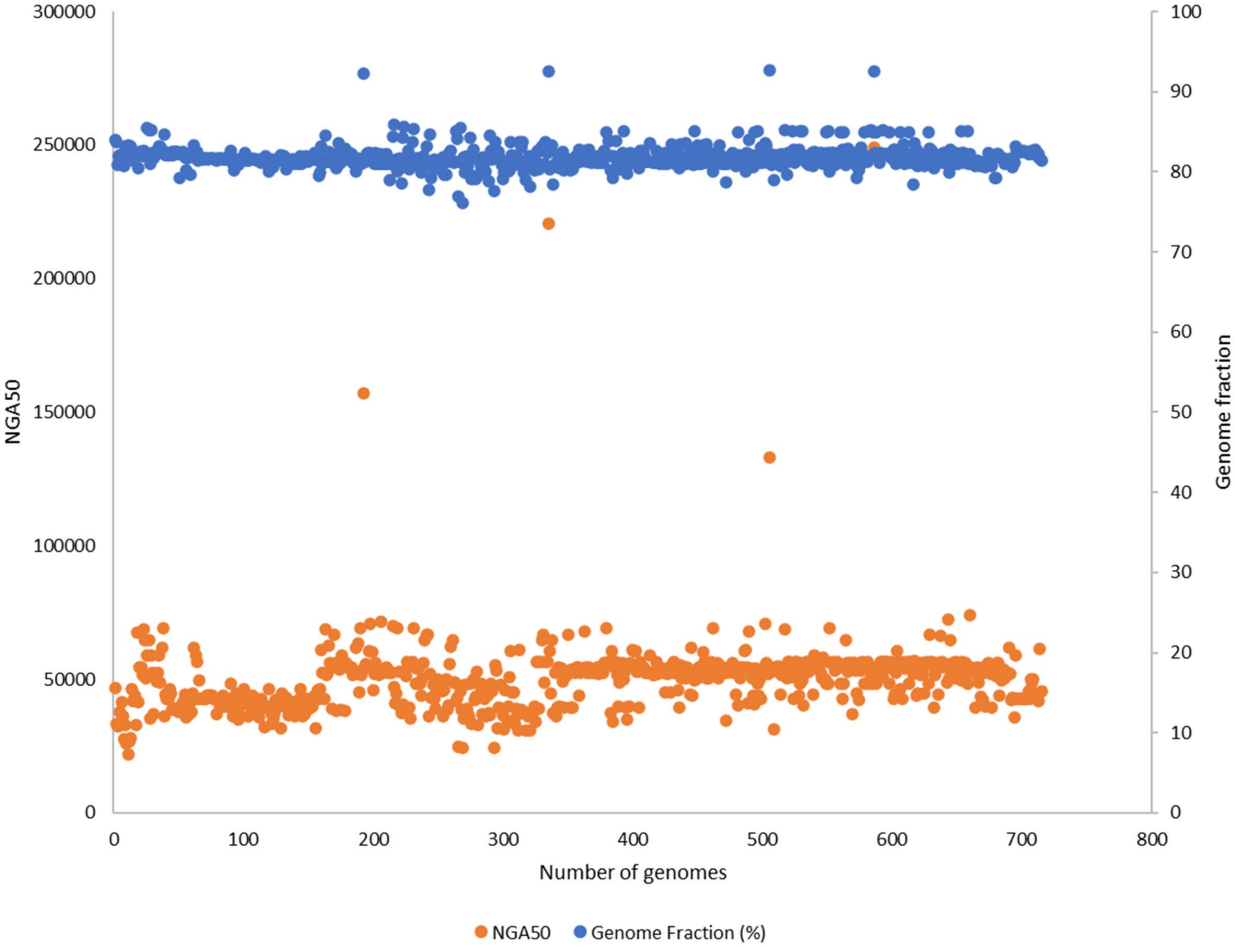
732 good quality genomes from PATRIC were subjected to quality metrics as defined by EUCAST (number of contigs <= 500, genome fraction >= 75%, NGA50 >= 20,000) and were used to identify good quality genomes for antibiotic susceptibility. 17 genomes did not qualify based on these criteria and were removed from subsequent analysis for implementing classification criteria.

Out of 732 good-quality genomes (as defined by PATRIC), CheckM contamination (≤5%) is reported for 34 and CheckM completeness (>=90%) is reported for 156 genomes, respectively (Figure 2B). These 732 good-quality genomes from PATRIC were subjected to quality metrics as defined by EUCAST (number of contigs <= 500, genome fraction >= 75%, and NGA50 >= 20,000) and are used to identify good-quality genomes for antibiotic susceptibility (Figure 5). Out of 732, 715 qualified as good quality genomes and the remaining 17 genomes did not qualify based on these criteria and were removed from subsequent analysis for implementing classification criteria (Supplementary File S3). Of these 715, 700 isolates have breakpoint classification as per CLSI and were used for further analysis.

### Strain classification

Phenotypic classification is performed on a total of 700 strains. Based on the same, 85 strains were classified as possible susceptible, 198 strains as possible MDR, 13 strains as possible XDR, and 404 strains could not be classified. None of the strains were classified as TDR.

When EUCAST:2022 breakpoints were given in the user defined breakpoint, 429 strains were classified as possible MDR and 271 strains were not classified. None of the strains were classified as susceptible, possible XDR, or TDR.

### Annotation for antimicrobial resistance genes

The genomes of the selected 700 isolates were screened for antimicrobial resistance using ABRicate. The results with detailed annotation of these antimicrobial genes are provided in Supplementary File S4. A total of 224 unique accession numbers of ARGs were mapped in these 700 isolates.

The tools and the workflows included in the Galaxy-ASIST enable non-bioinformatics researchers to perform analysis of the data generated after performing antimicrobial susceptibility testing without the need for any coding skills. All tools and their dependencies are installed on the Galaxy platform and are managed by the Conda framework for dependency management.

**Figure 6:**
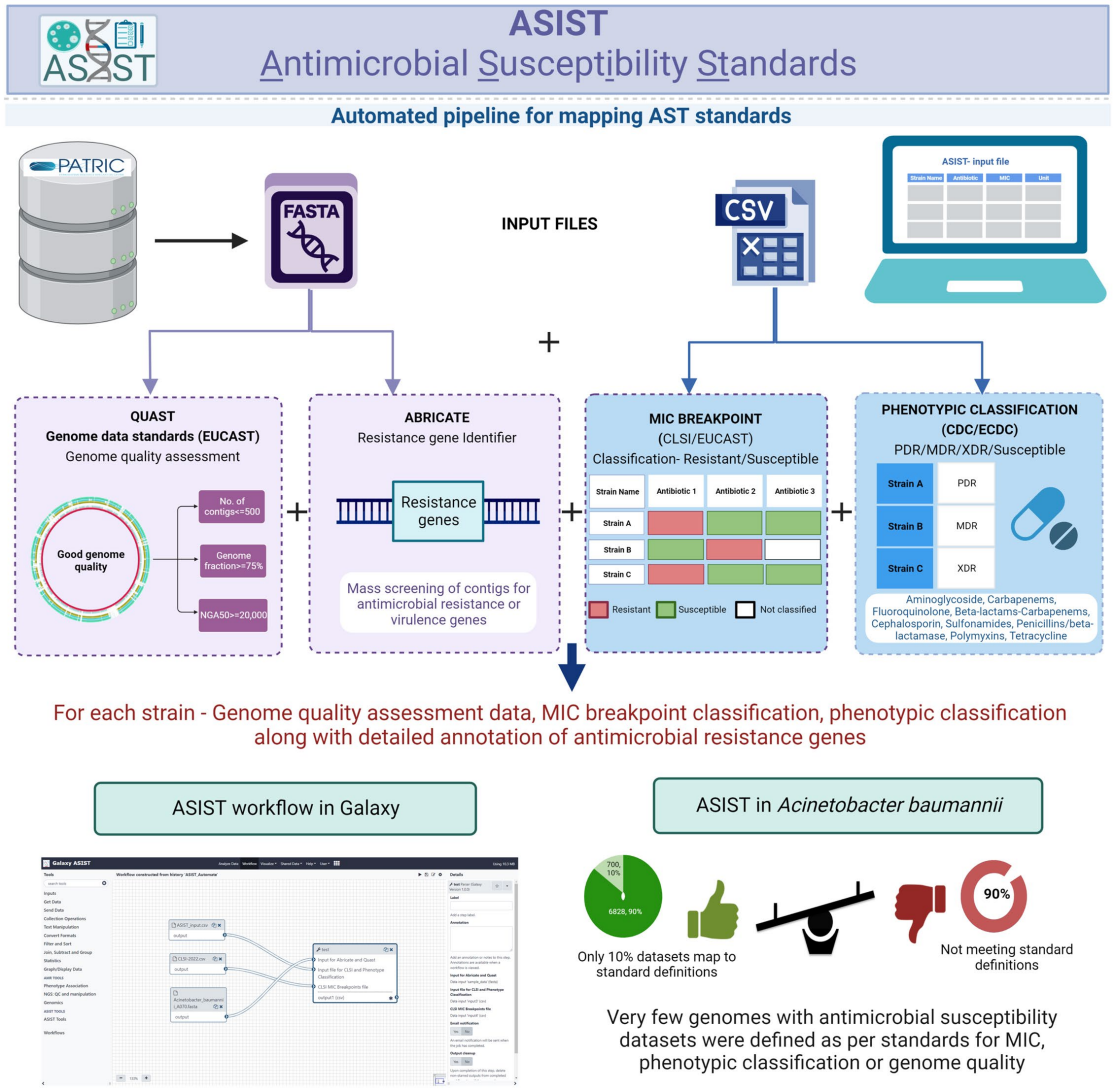
Galaxy-ASIST workflow. The existing modules along with the workflow interface are shown.

A graphical abstract depicting the overall workflow and the interface is shown in Figure 6.

## Discussion

Large-scale sequencing projects provide an opportunity to utilize WGS to augment *in vitro* AST as a part of routine laboratory diagnostic workflow, which guides the appropriate use of antimicrobials based on the drug susceptibility profile of pathogens. In the case of *Mycobacterium tuberculosis*, sequencing of isolates is proposed as the new gold standard for pan-susceptibility to first-line drugs and is being gradually adopted in clinical practice (Allix-Béguec et al., 2018). For some isolates, at least, the availability of MIC breakpoints is important for trough-level determination in the monitoring of the efficacy of some antibiotics (therapeutic drug monitoring and AUC dosing determination) in addition to the WGS since it enables the training of AMR reference databases. In addition, these two approaches may lead to the identification of novel AMR genes as well as the improvement of gene presence rules (Duggett et al., 2020). In order for WGS to be routinely used for establishing drug sensitivity profiles for clinical use, the adoption of standard practices as recommended by CLSI and EUCAST based on MIC breakpoints and assessment of genome quality is imperative (Magiorakos et al., 2012; Ellington et al., 2017). In recent years, several studies have discussed differences in these standards that impact clinical practice (Kassim et al., 2016; Cusack et al., 2019; Kahlmeter et al., 2019; Satlin et al., 2020). There are several open issues such as the use of “intermediate” for antibiotic susceptibility outcome, as it is argued that higher doses of antibiotics in these cases may lead to successful treatment but for clinical staff, the use of “intermediate” criteria may also indicate the antibiotic is ineffective (Kahlmeter et al., 2019). Given that the standards need to be harmonized globally (Turner et al., 2019), it is important to evaluate how they are being used in existing settings and what further gaps exist. To the best of our knowledge, none of the existing studies report an integrated analysis of the three global standards for MIC breakpoints, classification, and genome quality assessments. Toward this, we implemented these standards as a computational tool and have provided the assessment of the same with *Acinetobacter baumannii*.

As mentioned earlier, we have converged three global standards and observed that only 10% of all the data evaluated in this study had the metadata available for mapping to global standards. It is, therefore, recommended that the data reported from such studies should be taken into account for any genotype–phenotype correlations, as the phenotype defining criteria are uniform. In addition, MIC breakpoints are available for some antibiotics but are not used for the classification of MDR/XDR/PDR isolates. Therefore, interpretative criteria, which are defined for phenotype characterization of these strains, cannot be applied. Discrepancies in strain classification are also reported when CLSI and EUCAST breakpoints are used on the same isolates, which can influence the usage of antimicrobials (Suravaram et al., 2021). These observations highlight that standard harmonization is critical to extract maximum value from the data that are generated both for genotypic and phenotypic AST. There are several challenges to the implementation of these guidelines given that there are discrepancies in country-wise MICs with respect to breakpoints (Sánchez-Bautista et al., 2018; Standard Operating Procedures Bacteriology, 2019), and moreover, given that there are two standards, harmonization of standards depending on the evidence in a localized fashion is the need of the hour. Well-defined SOPs (for performing AST, genome sequencing, and mapping AMR determinants), their wider availability, and implementation will lead to uniform interpretative guidelines for susceptibility, improve reporting, and reduce variation of results between the laboratories. A global strategy is needed to reduce the variability of MICs across different settings and will significantly contribute to a better understanding of genotype-to-phenotype correlation for accurate estimations of susceptibility profiles as well as the discovery of novel drug-resistant determinants.

Although having implemented these standards, there are several other limitations such as hetero-resistance (Hong et al., 2020), media availability, or laboratory labeling error, concentration of ions, brand of antibiotics, and automation platform that may lead to variable MICs (Ezadi et al., 2019). These challenges limit the appropriate use of these antibiotics in clinical settings due to inconsistent AST. It is, therefore, strongly recommended that genome sequencing-based AST may be adopted widely for better classification of clinical isolates, as it offers sequence-directed therapies, a potential solution for universal susceptibility testing. Furthermore, it is also critical to evaluate the value of these susceptibility outcomes with respect to treatment success (Bader et al., 2018).

However, in resource-limited settings, the implementation of this method can be done starting with laboratories associated with tertiary care hospitals and medical colleges along with the upgradation of laboratory infrastructure and training of manpower. Collaboration with government set-up may help reduce the initial cost of implementation. The algorithm can later be expanded to include other pathogens of clinical significance along with the maintenance of region-specific repository.

The use of this platform along with active surveillance measures will help to flag danger sequences and prompt early action to curb the horizontal spread of AMR. In this study, we have designed an automated pipeline for assessing the data generated after antimicrobial susceptibility testing. Four tools have been customized in the Galaxy workflow system for performing the standard mapping followed by annotation of antimicrobial determinants as discussed earlier. This workflow system can be further expanded by incorporating other existing tools into the Galaxy Toolshed such as Prokka for annotating genes and identifying coding sequences in prokaryotic genomes, Roary for pan-genome analysis to identify core, accessory genes, unique genes (Seemann, 2014), etc.

Galaxy-ASIST is a web-based platform that allows for the characterization of clinical isolates based on three international standards. A comparative assessment is also performed on the two major standards for identifying resistant and susceptible isolates. In our case study, it is observed that despite the availability of several thousand genomes, nearly 10% are associated with metadata that allow for the interpretation of AST in globally acceptable standards. Harmonization of standards and their implementation in clinical practice is much needed. Galaxy-ASIST offers a strong foundation for seamless mapping of these standards on already available or new data generated and will contribute to the much needed structured metadata architecture to capture these standards, as they are revised from time to time so that genotype–phenotype correlations can be established for new strains and discovery of new resistant determinants may be quickly translated as markers of resistance in different clinical environments across the globe.

For applications involving very large datasets, the users may like to use a locally installed version of Galaxy-ASIST to achieve better performance as the genome sequence data upload will depend on the internet bandwidth at the users’ end. The study currently focuses on *Acinetobacter baumannii* but can be scaled for all priority pathogens in future versions of Galaxy-ASIST(GBD 2019 Antimicrobial Resistance Collaborators, 2022).

## Conclusion

Galaxy-ASIST offers a centralized repository and a structured metadata architecture to provide a single globally acceptable framework for AST profiling of clinical isolates based on global standards. The platform also offers subsequent fine mapping of antimicrobial-resistant genes. It is a user-friendly platform based on three global standards, MIC breakpoints, phenotypic classification, and genome quality, and is applied to the largest publicly available data (>6,500 whole-genome sequences) for *Acinetobacter baumannii* (AB). Galaxy-ASIST offers a strong foundation to capture these standards, as they are revised from time to time. ASIST is designed in a manner that makes it amenable to other priority pathogens too. This platform is expected to facilitate genotype and phenotype correlations for new strains to combat the increasing threat of AMR.

## Data availability statement

The original contributions presented in the study are included in the article/Supplementary material/Galaxy-ASIST platform, further inquiries can be directed to the corresponding author.

## Author contributions

AB conceived and designed the study. TS and SS performed data curation and analysis, and wrote the manuscript. JK and RK developed the Galaxy interface for ASIST. GB contributed to design and testing of the platform, including addressing reviewer concerns about the applicability of the platform in clinical settings. All authors contributed to the article and approved the submitted version.

## Funding

This work is supported by OLP1072 and HCP-0101.

## Conflict of interest

The authors declare that the research was conducted in the absence of any commercial or financial relationships that could be construed as a potential conflict of interest.

## Publisher’s note

All claims expressed in this article are solely those of the authors and do not necessarily represent those of their affiliated organizations, or those of the publisher, the editors and the reviewers. Any product that may be evaluated in this article, or claim that may be made by its manufacturer, is not guaranteed or endorsed by the publisher.
